# Edge-centric network control on the human brain structural network

**DOI:** 10.1162/imag_a_00191

**Published:** 2024-06-10

**Authors:** Huili Sun, Matthew Rosenblatt, Javid Dadashkarimi, Raimundo Rodriguez, Link Tejavibulya, Dustin Scheinost

**Affiliations:** Department of Biomedical Engineering, Yale University, New Haven, CT, United States; Department of Radiology, Athinoula. Martinos Center for Biomedical Imaging, Massachusetts General Hospital, Charlestown, MA, United States; Department of Radiology, Harvard Medical School, Boston, MA, United States; Interdepartmental Neuroscience Program, Yale School of Medicine, New Haven, CT, United States; Department of Radiology & Biomedical Imaging, Yale School of Medicine, New Haven, CT, United States; Department of Statistics & Data Science, Yale University, New Haven, CT, United States; Child Study Center, Yale School of Medicine, New Haven, CT, United States; Wu Tsai Institute, Yale University, New Haven, CT, United States

**Keywords:** edge-centric network, brain structure, network control, diffusion MRI

## Abstract

Network control theory models how gray matter regions transition between cognitive statesthrough associated white matter connections, where controllability quantifies the contributionof each region to driving these state transitions. Current applications predominantly adoptnode-centric views and overlook the potential contribution of brain network connections. Tobridge this gap, we use edge-centric network control theory (E-NCT) to assess the role of brainconnectivity (i.e., edges) in governing brain dynamic processes. We applied this framework todiffusion MRI data from individuals in the Human Connectome Project. We first validate edgecontrollability through comparisons against null models, node controllability, and structuraland functional connectomes. Notably, edge controllability predicted individual differences inphenotypic information. Using E-NCT, we estimate the brain’s energy consumption foractivating specific networks. Our results reveal that the activation of a complex, whole-brainnetwork predicting executive function (EF) is more energy efficient than the correspondingcanonical network pairs. Overall, E-NCT provides an edge-centric perspective on thebrain’s network control mechanism. It captures control energy patterns andbrain-behavior phenotypes with a more comprehensive understanding of brain dynamics.

## 1 Introduction

Structural connectivity of the white matter facilitates communications between gray matterregions (Yeh et al., 2021). Specifically, thisarchitecture supports the dynamics of switching between cognitive states (Sorrentino et al., 2021). Network control theory (NCT) provides atheoretical framework to quantify the energy needed for gray matter regions to switch betweenthese cognitive states (Gu et al., 2015). Thecontribution of each region to a state transition is known as “controllability”.Average controllability measures a region’s ability to drive transitions to nearbystates, while modal controllability measures a region’s ability to drive transitions tofar-away states. NCT has furthered our understanding of the brain across various neuroimagingstudies. For example, controllability develops rapidly in infancy (Sun, Jiang, et al., 2023) and continues gradually into adolescence(Tang et al., 2017) and adulthood (Gu et al., 2015). This change in controllability putatively underliesthe maturation of behaviors such as executive function (EF) (Cuiet al., 2020). Controllability is also altered in psychiatric (depression (Fang et al., 2021) and schizophrenia (Braun et al., 2021)) and neurologic (epilepsy (He et al., 2022) and Parkinson’s (Zarkali et al., 2020)) disorders. Finally, NCT provides a mechanistic understanding ofnoninvasive (neurofeedback) and invasive (transcranial magnetic stimulation) brain stimulationapproaches (Bassett & Khambhati, 2017).

Nevertheless, current brain-based applications of NCT are based on node-centric frameworks,which conceptualize the brain as a graph with regions as nodes and functional and structuralconnections as edges. This node-centric view overlooks potentially meaningful interactionsbetween edges (Faskowitz et al., 2022). Edge-centricframeworks instead extend the conventional concept of brain connectivity into higher dimensions,featuring interactions between the edges of a network instead of its nodes (Betzel et al., 2023). Through functional connectivity, individual-leveledge time series (Sporns et al., 2021) and edgecommunities (Faskowitz et al., 2020,^2021^;Gao et al., 2020) haveproduced complementary information to traditional node-centric graphs, including improvedindividual identifiability (Jo et al., 2021) and greaterstatistical power in network-level inference (Rodriguez et al.,2022). Several works from the edge-centric view have also shown promising results indetecting meaningful functional differences between study groups (Idesis et al., 2022;Jiang et al.,2022;Sun, Wang, et al., 2023;Yang et al., 2023;Zhang et al.,2023). In contrast to functional data, edge-centric communities based on structuralconnectivity have relied on group-averaged data due to a lack of time-series information used toconstruct edge-centric networks for an individual (de Reus etal., 2014). Individual-level edge-centric structural connectivity studies are not widelyinvestigated.

In this work, we use edge network control theory (E-NCT) to study how the structuralconnectome facilitates system-level dynamic brain activities. First, we demonstrate an efficientway to convert the standard node-centric structural connectome to an edge-centric network via anincidence matrix. This conversion enables us to apply NCT on edge networks at the individuallevel. To benchmark E-NCT, we characterize edge controllability against null models, compareedge controllability with node controllability, and contrast edge controllability to structuraland functional connectomes. To show the utility of E-NCT, we show that predictive models basedon edge controllability perform favorably compared with standard structural connectomes incapturing individual differences in age, intelligence quotient (IQ), and EF. Finally, wecalculate the energy cost necessary to change EF-related connectome patterns and compare it withthe energy cost for canonical brain networks. Together, we provide a novel conceptualization ofNCT for edge-centric brain networks. E-NCT can elucidate each edge’s role in supportingbrain dynamics and capture individual differences in complex phenotypes.

## 2 Methods

**Fig. 1.**
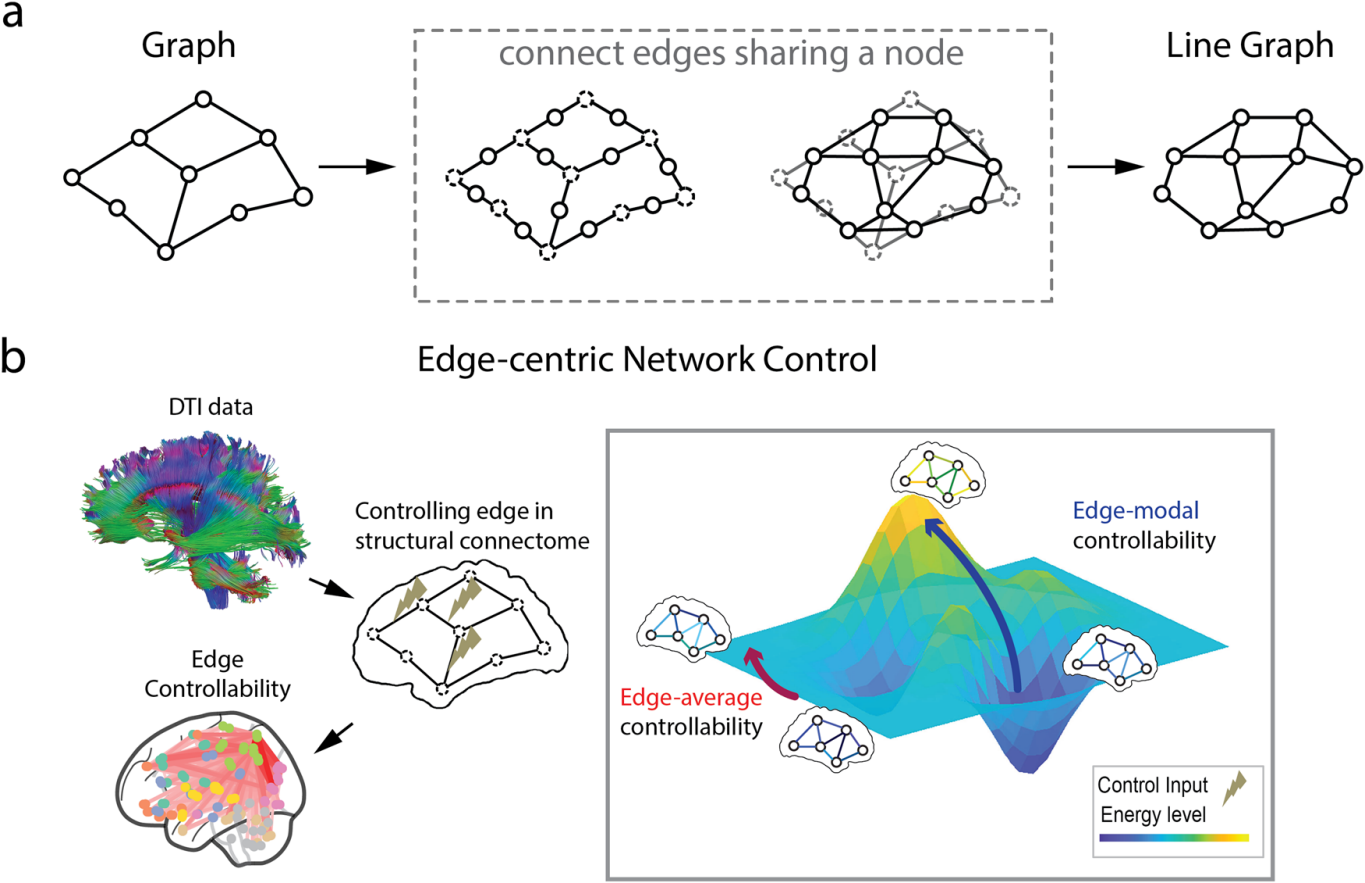
Edge-centric network control theory. (a) To create edge-centric brain networks, connectomeswere transformed from a graphG=[V,E]to a line graphL(G). (b) With DTI data, structural connectomes were constructed. By controllingedges through the network control framework, edge controllability is defined as how eachconnection in the brain contributes to brain state transitions.

Network control theory (NCT) aims to address the problem of how to control a complex networksystem through nodes (Liu et al., 2011) and edges (Nepusz & Vicsek, 2012). However, the classicapplication of network control theory on brain networks only focuses on measuring regionalcharacteristics (as nodes) while overlooking the role played by connectivity (as edges) inregulating system-level activities. Therefore, edge-centric network control theory (E-NCT) mayuncover distinct brain dynamics from controlling edges, further allowing the simulation of brainconnectomes (Fig. 1).

### 2.1 Network control theory

1. Dynamical process of brain activity
  To describe the dynamics of the neural system over time, NCT employs a simplified lineardynamic network model. In the continuous setting, this model is
$$\dot{\text{x}}\left(t\right)=\text{Ax}\left(t\right)+{\text{B}}_{K}{\text{u}}_{K}\left(t\right),$$ (1)
  wherexdenotes the brain state at a given time, and***A***is the symmetric, undirected, and weighted adjacency matrixfor the network. The input matrix${\text{B}}_{K}$identifies thecontrol points***K***in the system, where***K***= {k1,…,km} and${\text{B}}_{K}=\left[e_{k1}...e_{km}\right]$,$e_{i}$denotes the ithcanonical vector of dimensionNand input${\text{u}}_{K}$denotes the inputcontrol strategy over time. In the discrete setting, this model is$\text{x}\left(t+1\right)=\text{Ax}\left(t\right)+{\text{B}}_{K}{\text{u}}_{K}\left(t\right)$. The variables are analogous to the continuous setting. Consistent with theliterature, we define controllability (Deng et al.,2022;Gu et al., 2015) using the discretesetting and control energy using the continuous setting (Braunet al., 2021;Cui et al., 2020).
2. Average and modal controllability of each brain region
  Two metrics of controllability are defined to describe the ability to drive the networkwith different types of transitions as patterns of regional activity. Average controllabilitymeasures the ability to drive to nearby brain state transitions. Modal controllabilitymeasures the ability to drive to distant brain state transitions. Classic control theoryprovides that the controllability of the network from the set of network nodes***K***is equivalent to the controllability Gramian${\text{W}}_{K}$being inverted,where$W_{K}=\sum_{\tau =0}^{\infty }A^{\tau }B_{K}B_{K}^{T}A^{\tau }$for the discrete setting.
(a) Average controllability (AC)
  AC is defined by the average input energy from a set of control elements and overallpotential target states. The average input energy is proportional to$Trace\left({\text{W}}_{K}^{-1}\right)$,the trace of the inverse of the controllability Gramian. Here, to keep the consistency withprevious studies,$Trace\left({\text{W}}_{K}\right)$is used as themeasurement of average controllability to increase the accuracy of computation on small brainnetworks and maintain the information obtained by this measurement.
(b) Modal controllability (MC)
  MC is defined as the ability of an element to control difficult-to-reach modes of thedynamic network system and is computed from the eigenvectors$V=\left[v_{ij}\right]$of the adjacency matrix***A***. Here, we define the measurement ofmodal controllability as$\phi_{i}=\sum_{j=1}^{N}\left(1-\lambda_{j}^{2}\left(A\right)\right)v_{ij}^{2}$of allNmodes$\lambda_{1}\left(A\right),...,\lambda_{N}\left(A\right)$from brain regioni, following thedefinition of previous studies.
3. Control energy to activate brain regions
  To explore how the brain’s dynamic processes are constrained by the structuralconnectome, we model the brain system in the continuous setting and quantify the energyrequired to activate specific brain networks over the existing structural network topology ofthe brain. The baseline state**x**(0) was set to 0 to simulate the resting state ofthe brain, while the target brain state**x**(T) was defined such that all regionsin the desired brain network had a magnitude of 1 representing activation of the desiredregions. Following the definition of the control task in the previous studies (Cui et al., 2020;Gu,Betzel, et al., 2017), where the system transitions from initial state$\text{x}\left(0\right)={\text{x}}_{0}$to target state$\text{x}\left(T\right)={\text{x}}_{T}$with minimum-energyinput${\text{u}}_{K}\left(t\right)$as an optimalcontrol problem,
$$\begin{array}{l} \min_{u}{\int_{0}^{T}\left(x_{T}-x(t)\right)}^{T}(x_{T}-x(t))+\rho u_{K}(t^{T})u_{K}(t))dt,s.t.\\ \;\;\;\;\;\;\;\;\;\;\;\;\;\;\;\;\;\;\;\;\;\;\;\;\;\;\;\;x(t)=Ax(t)+B_{K}(t)u_{K}(t),x(0)=x_{0},x(T)=x_{T}, \end{array}$$ (2)
  whereTis thecontrol horizon,$\rho \in {\mathbb{R}}^{+}$,$\left({\text{x}}_{T}-\text{x}\left(t\right)\right)$is the distancebetween the state at timetand the targetstate.
  To solve the optimal control${\text{u}}^{*}$, we definethe Hamiltonian as
$$H\left(\text{p},\text{x},\text{u},t\right)={\text{x}}^{Τ}\text{x}+\rho {\text{u}}^{Τ}\text{u}+{\text{p}}^{Τ}\left(\text{Ax}+\text{Bu}\right).$$ (3)
  Based on the Pontryagin minimum principle (Boltyanskiy etal., 1960), if${\text{u}}^{*}$is an optimalsolution to the minimization problem with corresponding state trajectory${\text{x}}^{*}$, then thereexists${\text{p}}^{*}$such that
$$\frac{\partial H}{\partial \text{x}}=-2\left({\text{x}}_{T}-{\text{x}}^{*}\right)+{\text{A}}^{T}{\text{p}}^{*}=-{\dot{\text{p}}}^{*},$$ (4)
$$\frac{\partial H}{\partial \text{u}}=2\rho {\text{u}}^{*}+{\text{B}}^{T}{\text{p}}^{*}\;=0$$ (5)
  With the constraints fromeqs. 4and5, the optimization problem${\text{u}}^{*}$can be solved(Gu, Betzel, et al., 2017). Control energy for eachnode$k_{i}$was defined as$E_{k_{i}}\;=\int_{t=0}^{T}||{\text{u}}_{k_{i}}^{*}(\text{t})|{|}^{2}dt$, indicating the overall energy input required by the node to facilitate thedesired state transition.

### 2.2 Edge-centric network control

1. Transformation from the node adjacency matrixAto the incidencematrixC
  Due to the region-based nature of brain imaging, the node-centric brain networkGcan be directlyrepresented by anN×Nadjacency matrixA. This undirected and weighted network G can, therefore, be represented byincidence matrixC(Evans & Lambiotte,2009),
$$CC^{T}=A+D,$$ (6)
  whereAis thenode-centric adjacency matrix,Dis the node degreematrix with its diagonal elements as$d_{ii}=\sum_{\text{j}}{\text{A}}_{\text{ij}}$. TheN×Lincidence matrix C can be obtained as
$$C_{i\alpha }=\{\begin{array}{cc} \sqrt{A_{N_{ij}}} & \mathrm{if}\;\mathrm{node}\;i\;\mathrm{is}\;\mathrm{incident}\mathrm{with}\;\mathrm{edge}\;\alpha \\ 0 & \mathrm{otherwise}. \end{array}$$ (7)
2. Transformation from the incidence matrixCto the edgeadjacent matrix$A_{E}$
  To project the information of networkGinto a line graphL(G), the edge-centricadjacency matrix$A_{E}$can be obtainedby
$$A_{E}=C^{T}C-W,$$ (8)
  whereCis theincidence matrix andWis a diagonal matrix with each element as the weight of each edge. For each element in theedge-centric adjacency matrix,
$$A_{E_{\alpha \beta }}=\sum_{i}C_{ia}C_{i\beta }(1-\delta_{\alpha \beta }),$$ (9)
  where C is the incidence matrix and$\delta_{\alpha \beta }=1$ifα=β.
3. Calculate controllability for each edge fromSection2.1. Part 2 based on edge-centric network
  Based on the edge-centric brain network, represented by edge adjacency matrix$A_{E}$, the input vector${\text{u}}_{L}$injects energy onthe edges to drive the network from its initial state to a targeted state. Therefore, theedge-centric adjacency matrix of the structural connectome for each individual functions asthe wiring diagram in the linear brain state transition. The input matrix${\text{B}}_{L}$is inL×1dimension, with each element indicating if the edge receives control input.Combining the controllability definition fromSection2.1. Part 2, average controllability for each edge (eAC) is defined as$Trace\left({\text{W}}_{L}\right)$, where$W_{L}=\sum_{\tau =0}^{\infty }A_{E}^{\tau }B_{L}B_{L}^{T}A_{E}^{\tau }$and modalcontrollability of each edge (eMC) equals$\sum_{\beta =1}^{N}(1-\lambda_{j}^{2}(A)v_{E\alpha \beta }^{\;\;\;2}$,where$V_{E}=[v_{E\alpha \beta }]$are the eigenvectors of edge-centric adjacency matrix$A_{E}$and$\lambda_{E}$are thecorresponding eigenvalues.
4. Adapt control energy to control a connectome fromSection2.1. Part 3
  Control energy$E_{L}$is available fromsolving the optimal control problem
$$\begin{array}{l} \min_{u}{\int_{0}^{T}\left(x_{T}-x(t)\right)}^{T}(x_{T}-x(t))+\rho u_{L}(t^{T})u_{L}(t))dt,s.t.\\ \;\;\;\;\;\;\;\;\;\;\;\;\;\;\;\;\;\;\;\;\;\;\;x(t)=A_{E}x(t)+B_{L}(t)u_{L}(t),x(0)=x_{0},x(T)=x_{T}, \end{array}$$ (10)
  and summarizing the optimal input energyu*over time:${\text{E}}_{L}\;=\int_{t=0}^{T}||{\text{u}}_{l}^{*}\left(t\right)|{|}^{2}\;dt$.

Following the parameter setting of the previous study, we used T=1 with step size of0.001 for the time horizon of control. Therefore, there were 1000 steps for the system to getthe target state from the initial state during simulation. Here, the target states were definedas 28 inter-/intra- networks of canonical networks (i.e., visual, somatomotor, dorsalattention, ventral attention, limbic, frontoparietal, and default mode networks). Edges in thetarget network were assigned a value of 1, indicating activated. All other edges were assigned0’s, indicating not activated.

### 2.3 Datasets

Two datasets were used in this study. Primary analyses were conducted on data from the HumanConnectome Project Young Adult (HCP-YA;https://www.humanconnectome.org/study/hcp-young-adult) and replicated on data from theHuman Connectome Project Development (HCP-D;https://www.humanconnectome.org/study/hcp-lifespan-aging), which includes only theadult part (age>18 years) of participants.

#### 2.3.1 HCP-YA

The HCP-YA imaging protocol details have been extensively documented (Glasser et al., 2013). In summary, all MRI data were obtained with a3T Siemens Skyra using a slice-accelerated, multiband, gradient-echo, EPI sequence (72 slicesacquired in the axial-oblique plane, TR = 720 ms, TE = 33.1 ms, flipangle = 52°, slice thickness = 2 mm, in-plane resolution= 2 mm × 2 mm, multiband factor = 8) and a MPRAGE (256 slices acquired inthe sagittal plane, TR = 2400 ms,TE = 2.14 ms, flip angle = 8°, slicethickness = 0.7 mm, in-plane resolution = 0.7 mm ×0.7 mm).

Diffusion MRI was sampled using a multishell diffusion scheme with a maximum b-values of3000 s/mm^2^, in-plane resolution of 1.5 mm, and slice thickness of 1.5 mm. Thediffusion data were reconstructed using generalized q-sampling imaging (Yeh et al., 2010) with a diffusion sampling length ratio of 1.25. Thetensor metrics were calculated and analyzed using the resource allocation (TG-CIS200026) atExtreme Science and Engineering Discovery Environment (XSEDE) resources (Towns et al., 2014). Whole-brain fiber tracking was conducted withDSI-studio with quantitative anisotropy (QA) as the termination threshold. QA values werecomputed in each voxel in their native space for each subject. These QA values are used towarp the brain to a template QA volume in Montreal Neurological Institute (MNI) space usingthe statistical parametric mapping (SPM) nonlinear registration algorithm. Once in MNI space,spin density functions were again reconstructed with a mean diffusion distance of 1.25 mmusing three fiber orientations per voxel. Fiber tracking was performed in DSI studio with anangular cutoff of 60°, step size of 1.0 mm, minimum length of 30 mm, spin densityfunction smoothing of 0.0, maximum length of 300 mm, and a QA threshold determined by DWIsignal. Deterministic fiber tracking using a modified FACT algorithm was performed until10,000,000 streamlines were reconstructed for each individual. Here, we used AAL2 atlas (Rolls et al., 2015) in MNI space with 120 nodes to constructindividual structural connectome. The pairwise connectivity strength was calculated as theaverage QA value of each fiber connecting the two end regions. Edges with a value less than0.001 were interpreted as not being connected and having a value not equal to 0 due tonumerical instability in the fiber tracking algorithm, and, therefore, set to 0. This processresulted in a 120 x 120 matrix for each participant.

The preprocessing of resting-state functional MRI data from HCP-YA followed the steps fromprevious studies (Dadashkarimi et al., 2023;Greene et al., 2018). The HCP minimal preprocessing pipelinewas used for artifact removal, motion correction, and registration. All subsequentpreprocessing was performed in BioImage Suite (https://bioimagesuiteweb.github.io/)and included standard preprocessing procedures, including removal of motion-related componentsof the signal; regression of mean time courses in white matter, cerebrospinal fluid, and graymatter; removal of the linear trend; and low-pass filtering. Like the structural connectomeconstruction, the AAL2 atlas was applied to the preprocessed fMRI data to create a mean timeseries for each brain region. Functional connectomes were then generated by calculating thePearson correlation between each pair of node-wise time series and then taking the Fishertransform.

We restricted our analyses to those subjects who participated in all fMRI and dMRI scans,whose mean frame-to-frame displacement during fMRI was less than 0.1 mm, and for whom IQmeasures and executive function task (i.e., List Sorting, Card Sorting, and Flanker task)scores were available (n = 515; 241 males; ages 22–36+ years).

#### 2.3.2 HCP-D

HCP-D imaging protocol details are documented bySomervilleet al. (2018). Here, we only included 149 subjects over age 18 years for replicationand validation. Demographic information is summarized inTable S1. All HCP-D brain imaging isobtained from 3T Siemens Prisma scanners. Diffusion imaging samples 185 directions on 2 shellsof b = 1500 and 3000 s/mm^2^, along with 28 b = 0 s/mm^2^images. Construction of structural connectomes with the diffusion data used the same pipelineas above in HCP-YA. The diffusion data were reconstructed using generalized q-sampling imagingwith quantitative anisotropy (QA) as the termination threshold. The AAL2 atlas with 120 nodeswas used to construct an individual structural connectome with averaged QA values from fiberstracked between two regions, resulting in a 120 x 120 adjacency matrix for eachparticipant.

### 2.4 Null model

To validate specificity of our controllability results, we constructed 5000 null models forthe group-averaged structural connectome by randomly rewiring connections from the originalnetwork with weight, degree, and strength distribution (Rubinov& Sporns, 2010). We calculated the corresponding controllability measures (i.e.,control energy, average controllability) for each rewired connectome. The number of times thatempirical controllability is higher/lower than the permuted controllability provides anonparametric p-value. When appropriate, multiple comparisons were corrected with falsediscovery rate (FDR).

### 2.5 Connectome-based prediction model

We used connectome-based predictive modeling (CPM) to test edge controllability’sability to predict individual phenotypes. Age, fluid intelligence (IQ), and three EF tasks werepredicted. We also predicted a composite score of the three EF tasks based on a principlecomponent analysis (PCA) of the tasks. The literature routinely uses these phenotypes forbenchmarking predictive models (Butler et al., 2014;Litwińczuk et al., 2022;Schumacher et al., 2019). Independently, node controllability(including both AC and MC in a single model), structural connectivity, eAC, and eMC were theinput features for CPM. We compared prediction performance from edge controllability to nodecontrollability and structural connectivity.

We used CPM with a feature selection threshold at p = 0.01 (Shen et al., 2017). Tenfold cross-validation was performed. The datasetwas randomly divided into 10 subgroups, among which 9 subgroups were used as training and theleft 1 as a testing group. Model training involves feature selection of relative brain featureswithin the training group using Pearson’s correlation. The brain features (e.g., nAC/nMCfor each node or eAC/eMC/edge strength of SC for each edge) significantly correlated (p-value< 0.01) with the phenotype measure (e.g., EF, IQ, or age) were retained. Next, theselected features for each individual were summarized into a single number. Linear regressionwas then used to model this summary score and the phenotype in the training group. Finally,this model was applied to the testing group. This process was repeated iteratively, with eachsubgroup being the testing group once, generating a predicted result for each individual in thedataset. We predicted each phenotype independently (EF, IQ, and age). To compare the predictionperformance across different brain features, we performed 1000 repeats of 10-foldcross-validation in the same train-test group across the different brain features. p-Valueswere determined by the proportion of predictions for edge controllability that were better thanthat of other features among the 1000 repetitions.

## 3 Results

### 3.1 Edge controllability distribution across whole brain

The whole-brain distribution of edge controllability is shown inFigure 2a. Edges with the highest average controllability were observed withinthe parietal lobe and between the parietal and occipital lobes. Edges with the highest modalcontrollability are observed within and between the temporal lobe, cerebellum, and subcorticalregions. We define eAC_whole-brain_and eMC_whole-brain_as the sum of eAC oreMC across all edges. Across individuals, eAC_whole-brain_andeMC_whole-brain_are negatively correlated (r = -0.81, p<0.001),suggesting that individuals with better controllability on nearby-state transitions tend todisplay less capability to control distant brain state transitions. There were no sexdifferences in eAC_whole-brain_(t = -0.86, p = 0.38) andeMC_whole-brain_(t = 0.71, p = 0.47). For each edge, eAC is negativelyassociated with eMC across all individuals (r = -0.93, p<0.001), suggesting anedge can only control nearby or distant state transitions. These results are consistent withprevious node controllability results (Gu et al.,2015).

**Fig. 2.**
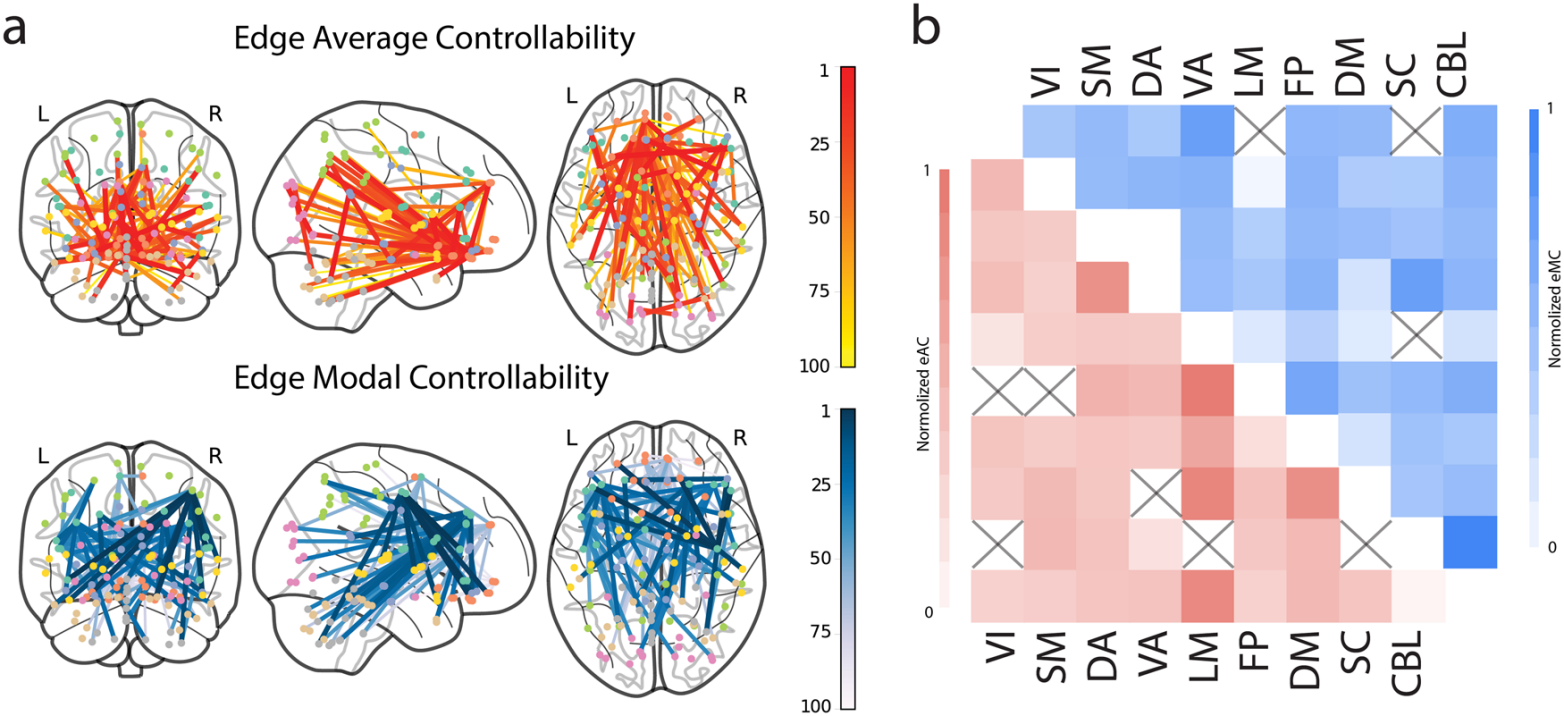
Edge controllability distribution. (a) Edges with the top 100 largest eAC (red on the left)and eMC (blue on the right) values. (b) Heatmaps of normalized within and between networkedge average (lower triangular matrix with red) and modal controllability (upper triangularmatrix with red) (box crossed: edge controllability in the empirical brain network is notsignificant from that of null models). VI=visual, SM=somatomotor,DA=dorsal attention, VA=ventral attention, LM=limbic,FP=frontoparietal, DM=default mode, SC=subcortical, andCBL=cerebellar networks

To investigate the specificity of our results, both measures of edge controllability weresignificantly different than the null models on the whole-brain level (Fig. S1). eAC_whole-brain_inthe real network was significantly lower than in the null models (p<0.001). Theseresults may be caused by the decrease of rich clubs and increase in local connections duringthe rewiring step in creating null models (Rubinov &Sporns, 2010;Van Den Heuvel and Sporns, 2011).This topology leads to the null models that are more capable to drive short-distance systemtransitions on the energy landscape (i.e., increased eAC_whole-brain_).eMC_whole-brain_in the real networks was significantly higher than in the nullmodels ( p<0.001), suggesting that the structural connectome’s rewiring leads toless efficient, long-distance state transitions. After correcting for multiple comparisonsusing FDR, 4259 edges had an average controllability significantly lower than the null model.In total, 4517 edges had a modal controllability significantly higher than the null models.This corresponds to 59.6% and 63.3% of edges exhibiting significantly different controllabilitythan the null models. The distribution of significant edges is shown on network level inFigure S2, highlighting the large numberof edges in the cerebellar and limbic networks.

We also investigated edge controllability for nine canonical networks by summing eAC and eMCover all edges in a canonical network. A critical advantage of edge controllability is thatcontrollability can be separated into within-network (e.g., the controllability of edges in thedefault mode network—DMN) and between-network controllability (e.g., the controllabilityof edges between the DMN and the frontoparietal network—FPN). Edges with highest eAClocated within the limbic network and between limbic and somatosensory, and default modenetworks. Edges with highest eMC lived within the cerebellum, and between ventral-attention andsubcortical networks (Fig. 2b).

Separating controllability into within-network (e.g., the controllability of edges in theDMN) and between-network components via E-NCT allowed us to observe these reversedpatterns.

### 3.2 Consistency and distinctiveness of edge controllability

**Fig. 3.**
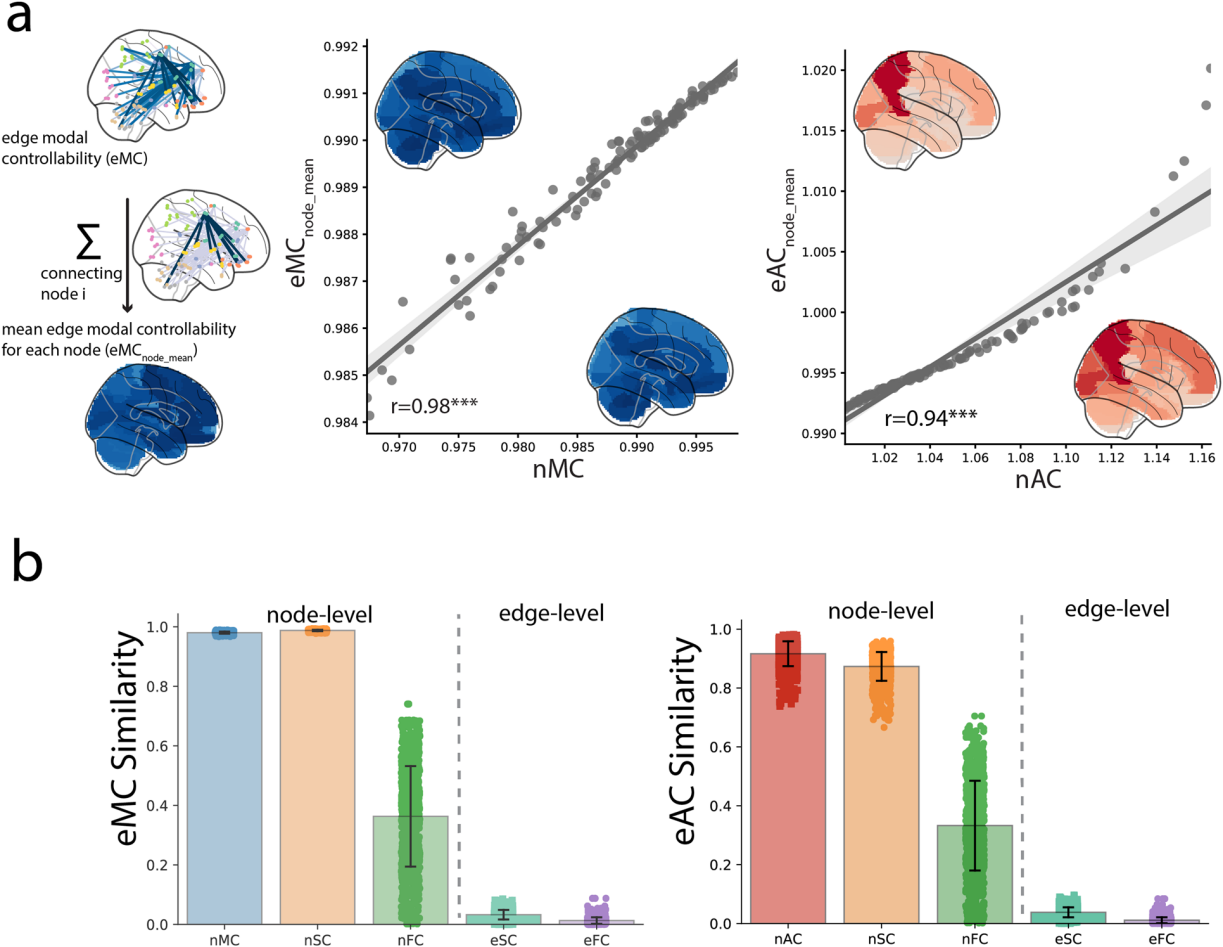
Relationships between edge controllability and conventional brain network measurement. (a)Mean edge controllability for each node (eAC_node-mean_; eMC_node-mean_) isthe sum of the controllability for edges connecting to the node. On the group level,eAC_node-mean_and eMC_node-mean_are highly correlated with nAC and nMC,respectively. Normalized eC_node-mean_and node controllability are shown on brainmaps. (b) For each individual, both edge average and modal controllability showed highsimilarity (measured as the absolute value of Pearson’s correlation r-value) with nodecontrollability and node degree of structural/functional connectome but not the edge strengthof structural/functional connectome.

To provide face validity of edge controllability, we tested the consistency betweencontrollability based on node-centric and edge-centric networks. First, we summed the edgecontrollability of every edge linked to one node and divided it by the number of edges,resulting in the mean edge controllability for each node. We labeled theseeAC_node-mean_and eMC_node-mean_.On the group level, node controllabilityand node-wise mean edge controllability are strongly correlated (Pearson’s correlation;node AC and eAC_node-mean_: r = 0.94, p<0.001; node MC andeMC_node-mean_: r = 0.98, p<0.001;Fig.3a).

Moreover, eAC_node_mean_was positively correlated with the node strength (r= 0.91) and eMC_node_mean_was negatively correlated with the node strength (r= -0.99) in the structural connectome (nSC); eAC_node_mean_was correlated withthe positive node strength (r = 0.20) and negative node strength (r = -0.43), andeMC_node_mean_was correlated with the positive node strength (r = -0.29) andnegative node strength (r = 0.52) in the functional connectome (nFC). These resultssuggest that edge controllability measures information similar to conventional brain networkmeasurements for nodes when summarized at the node level.

However, edge controllability revealed distinctive information on the edge level comparedwith edge features from structural and functional connectomes. For structural connectomes, onthe edge level, edge strength showed weak correlations with eAC (r = -0.06) or eMC (r= 0.05). Similarly, for functional connectomes, edge strength showed no significantcorrelations with eAC (r = -0.005) or eMC (r = 0.02) (Fig. S3). Further, eAC (r = 0.03)and eMC (r = -0.03) were not strongly correlated with the Euclidean distance betweennodes. In contrast, SC (r = -0.20) and FC (r = -0.45) showed typical correlationswith the Euclidean distance between nodes (Fig. S4). Overall, we showed that edge controllability remained consistent withconventional nodal measurements on the node level but allowed information to be investigated ata finer grain on the edge level (Fig. 3b).

### 3.3 Edge controllability better predicts behavior

**Fig. 4.**
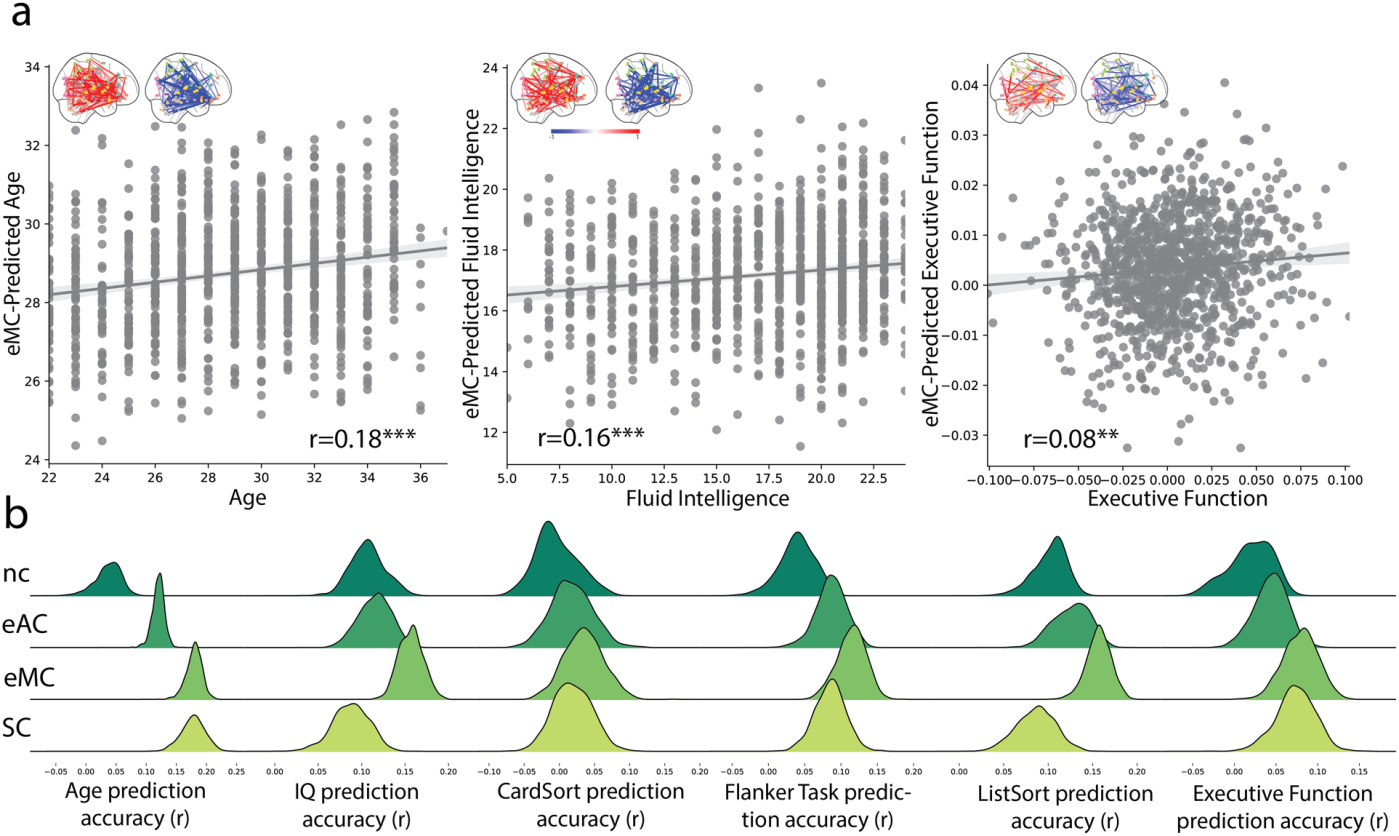
Prediction of individual phenotypes with edge controllability. (a) Scatter plots showingthe predicted values from models based on eMC compared with observed values for age, IQ, andEF. **p<0.01, ***p<0.001 (b) Distributionsof r-value of Pearson’s correlation between predicted scores and behavioral scoresiterated 1000 times for each task with structure-based brain features (i.e., nodecontrollability, edge average/modal controllability, and structural connectivity)

Numerically, eMC models exhibited the strongest correlation between observed and predictedphenotypes (Fig. 4a;Table S2). Among all predictable tasks,edge controllability outperformed node controllability, particularly in the Flanker test andthe executive function principal component, for which reliable predictions could not begenerated based on node controllability (Fig. 4b).Additionally, for the fluid intelligence scores (p = 0.001) and list sorting (p =0.003) scores, the prediction with edge modal controllability was significantly higher thanthose of structural connectivity. Card sorting scores cannot be predicted from the brainfeatures investigated here. Additional prediction results are shown inTable S2, including prediction fromfunctional connectivity and controlling feature dimensionality. Node controllability has 120features, whereas all other data have 7140 features. CPM models were rerun limiting the numberof features to 120. The prediction results were replicated with kernel ridge regression asshown inTable S3.

### 3.4 Energy-efficient activation of the EF-related functional network

Previous NCT work has shown that the energy cost of transition to the activation state for aparticular network relates to behavioral and developmental patterns. For example, the energeticcost to activate the FPN decreases over adolescence, allowing for improved EF as one becomes anadult (Cui et al., 2020). We extend this approach beyondnode activation to understand the energy cost of changing the strength of functionalconnectivity.

**Fig. 5.**
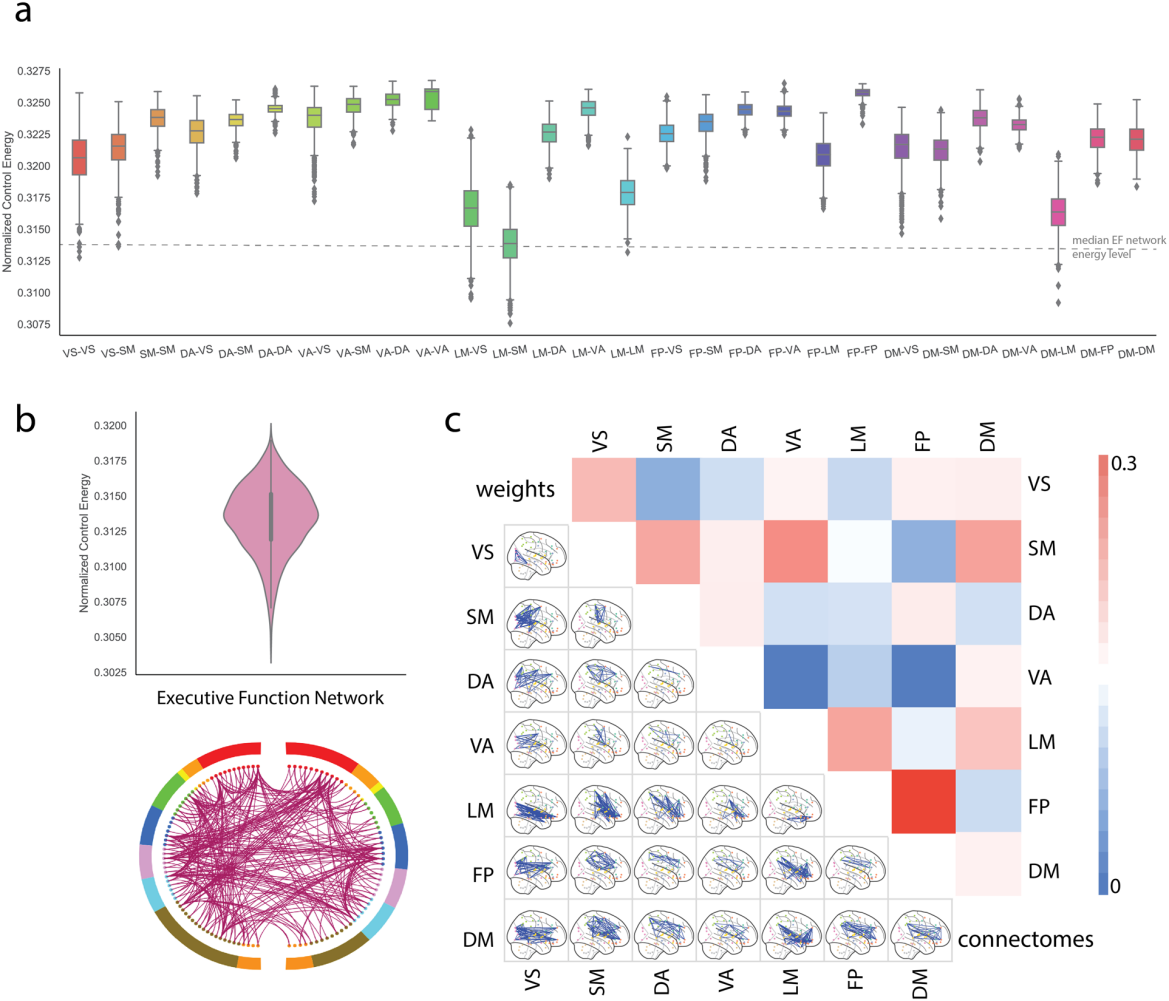
Simulation of the EF-related functional network. (a) Normalized control energy needed tochange connectivity in 28 canonical network pairs (7 within- and 21 between-networks). Allbut the dorsal attention network were significantly different than null models that preservedweight, degree, and strength distribution. (b) An EF predictive network was identified with aconnectome-based predictive model using resting-state fMRI data. Like most predictive models,the EF network was complex, consisted of 100’s of edges, and spanned across the sevencanonical networks. However, the normalized control energy needed to change connectivity inthe EF predictive network was lower than in any canonical network. (c) Energy used to changethe EF predictive network was decomposed into energy used by the 28 canonical network pairs.Within FPN, edges required the most energy to change.

We calculated the energy cost necessary to change connectivity within and between canonicalbrain networks using E-NCT. Similar to previous control energy studies, edges in the targetnetwork (i.e., 1 of the 28 between/within networks) were assigned a value of 1. Edges in everyother network were assigned a value of 0. Normalized network control energy for each edge-levelcanonical network, defined as the control energy to change the target network divided by thenetwork size (i.e., number of edges in the network;Fig.5a), was significantly higher than those of null models except edges within the dorsalattention network (Fig. S5).Consistent with node-centric results (Cui et al., 2020),changing the edges within the FPN requires the highest amount of control energy. However, someedges to the FPN, like edges between the FPN and limbic networks, were recruited withsubstantially less energy to change. Together, these results suggest that the high cost toactivate FPN nodes is driven by within FPN connections rather than connections between othernetworks. The network pairs that included the limbic network were the easiest to change.

We also investigate the cost of changing connectome patterns in complex, whole-brain networksfrom predictive modeling. First, we used resting-state functional connectomes to predict EFusing CPM with 10-fold cross-validation and a feature selection threshold of p<0.01. Weobserved significant prediction performance (r = 0.11, p = 0.018). Next, wecalculated the required energy to change the connectivity of this predictive network as definedby the positive network from CPM. Similar to the above, the magnitude of the edges in thepositive network from CPM (Fig. 5b) was assigned 1, andall other edges were set to 0. Surprisingly, the energy cost to change the EF predictivenetwork is lower than any within or between network connectivity. This suggests that the energyto change complex, predictive networks—which putatively better reflect the underlyingbehavior—might be lower than that to change canonical networks. Next, we further brokedown the energy cost to change the EF network by canonical networks on the population level.Edges within the FPN mostly explain the EF network’s energy costs and not connectionsbetween the FPN and other networks (Fig. 5c). Further, foreach individual, differences in contributions of canonical network control energy for the EFnetwork can be associated with their fluid intelligence performance. The energy contributionsof FP-VS (r = -0.095, p = 0.028, FDR-corrected) and VA-LM (r = -0.087, p= 0.045, FDR-corrected) were negatively correlated with individual fluid intelligencescores, while those of DM-DM were positively correlated with intelligence (r = 0.10, p= 0.024, FDR-corrected;Fig.S6). This result suggested that—in addition to the total energyconsumption—the personal strategy of using control energy to change brain networks mightbe related to individual cognition performance.

### 3.5 Replication of E-NCT in external data

Finally, we replicated our E-NCT results in a secondary dataset using young adults(>18 years old; n = 149) from the HCP-D dataset. As shown inFigure S7, edge controllability betweenthe two datasets was highly correlated (eAC: r = 0.93; eMC: r = 0.93), indicatinghigh consistency between which edges exhibited large edge controllability across datasets.Additionally, associations between edge controllability and node controllability, structuralconnectivity, and functional connectivity also hold in the replication from the HCP-D dataset(Table S4).

## 4 Discussion

Research in network science has advanced in descriptions of the structural and dynamicproperties of networks, on both nodes (Liu et al., 2011)and edges (Nepusz & Vicsek, 2012;Pang et al., 2017). Integrating network control theory (NCT) andedge-centric brain networks, we use edge-centric NCT (E-NCT) to calculate the controllabilityfor each edge (i.e., edge controllability) in the connectome. We converted the standardnode-centric connectome into an edge-centric network using an incidence matrix to createedge-centric networks from structural data for each individual. This step enabled theapplication of E-NCT for each individual. To demonstrate the framework’s effectiveness,the analysis of edge controllability on null models provided evidence that edge controllabilitycould reflect the topological characteristics of structural connectomes. Furthermore, thevalidity of edge controllability was tested by mapping the edges back to each node and comparingit with node controllability. Additionally, edge controllability predicted individualdifferences in phenotypic information. Finally, simulating control energy to change edges thatpredict EF revealed a distinct energy consumption pattern from canonical networks. Byconsidering the interactions between edges, E-NCT naturally distributes a node’scontrollability among its edges, distinguishing the contribution of network-to-networkcollaborations and giving a more comprehensive view of brain dynamics.

When summing the edge controllability of each edge connected to a node, we found a highconsistency with traditional node controllability. This result suggests that E-NCT allows us tobreak down how each edge contributes to a node’s controllability. The consistency betweenedge and node controllability also suggests that traditional node controllability can berecovered from edge controllability. Computationally, one could explicitly calculate only edgecontrollability and recover node controllability rather than calculate both independently. Thisassociation between edge and node controllability provides an intuitive interpretation. Nodecontrollability is proportional to summing edge controllability for edges belonging to thatnode—analogous to node degree and strength. Nevertheless, this result is expected (Novelli & Razi, 2022) because the same structuralconnectome is used to estimate both controllability measures. Overall, edge-centric methodsoriginate from the same information source but at a finer resolution than node-centricmethods.

Furthermore, moving from node to edge controllability benefitted downstream analyses. Edgecontrollability showed an improved prediction of phenotypic information over nodecontrollability. One explanation is that brain features on the edge level include considerablygreater information than those on the node level, with complexity in the order of$O(n^{2})$for edges compared withO(n)for nodes. Thisinterpretation aligns with other works showing that connectomes achieve better predictions thanregional approaches (Jo et al., 2021;Sun, Wang, et al., 2023;Zhang et al.,2023). Additionally, as node controllability appears to be proportional to the sum ofedge controllability, this summing averages out effects, reducing information for prediction.Analyzing controllability at the edge level retains this information, which could be necessaryto better understand cognition or psychiatric and neurological disorders in future applicationsof E-NCT.

A further example of this benefit is seen in the energy consumption simulations, where theenergy cost of complex, whole-brain task-predictive networks can be simulated and broken down bycanonical network pairings. This advance is essential as many behavioral phenotypes manifestthrough the interaction between multiple networks and regions (Mišić & Sporns, 2016). As such, connectome-based predictive modelsbetter predict and, putatively, better reflect the underlying functional anatomy of cognition(Avery et al., 2020;Suiet al., 2020) and clinical disorders (Garrison et al.,2023;Greene & Constable, 2023;Vogel et al., 2023) than models involving only functionalspecific regions. Our preliminary results suggest that these whole-brain task-predictivenetworks are easier to change than canonical brain networks and may better reflect the energeticcosts of behaviors such as EF. Additionally, for the EF network, changing the connectivity statefor edges within the FPN required the most energy, consistent with control energy studies with anode-centric approach (Cui et al., 2020). This highcontrol energy cost was not observed in connections between the FPN and other networks. Thesefindings are consistent with the FPN being a flexible hub (Adeliet al., 2019) that changes its connectivity to other networks to switch betweendifferent tasks—a component of EF. Calculating control energy for within- andbetween-network edges independently exhibits how the FPN can have high internal activation costswhile being highly adaptive in its connectivity to other networks.

NCT promises to explain and predict how one can exogenously steer the brain away frompathological states and toward healthy states (Muldoon et al.,2016). Although node-centric NCT is currently used in brain stimulation studies (Hahn et al., 2023), growing noninvasive and invasive brainstimulation approaches have been moving from targeting a single region to multiple regions andtheir connections. For example, connectome-based neurofeedback has shown promise in targetingthe connectivity of edges (Scheinost et al., 2020). Thefield of deep brain stimulation also is shifting from targeting anatomical regions towardactivation of white matter pathways (Choi et al., 2021).For example, full activation of white matter pathways predicted better treatment response todeep brain stimulation of the subcallosal cingulate for treatment-resistant depression (Song et al., 2023). Highly controllable edges may be betterwhite matter pathways to activate. Additionally, E-NCT could potentially serve as acomputational model to guide transcranial electrical stimulation (Woods et al., 2016), such as transcranial direct current stimulation(tDCS) and transcranial alternating current stimulation (tACS). Highly controllable edges couldbe targeted by placing anodes and cathodes at the two ends of those edges.

Our work has several strengths. Previous work has investigated edge-centric functionalnetworks using their temporal characteristics (Sporns et al.,2021) to study the network features such as community structures (Chumin et al., 2022;Idesis et al.,2022) and modularity (Gu, Yang, et al., 2017) onthe individual level. However, edge-centric structural connectomes have primarily been studiedat the group-averaged level (de Reus et al., 2014) ratherthan at the individual level. This limitation arises from the difficulty in constructing anedge-centric connectome for each individual. First, we efficiently construct edge-centricconnectomes for each individual utilizing the incidence matrix transform between a graph(node-centric network) and a line graph (edge-centric network). Second, eMC and eAC were uniquein the edge strengths of both structural and functional connectomes. However, when summing edgestrength over all edges connected to a node (i.e., the node level), eAC_node_mean_andeMC_node_mean_were strongly correlated with equivalent measures from structural andfunctional connectomes. Other real-world networks, including the US telecommunication networkand most World Wide Web networks, also show significant differences when using an edge-centricview (Nepusz & Vicsek, 2012), mainly due to thehighly hierarchical structure of the networks (Battiston et al.,2021). Third, we compared edge controllability with null models, preserving thenetwork’s degree distribution, and observed that the edge controllability differedsignificantly from that of null models. Additionally, the effect of rewiring during null-modelreconstruction is hierarchically distinct for each edge. The higher the controllability in thenetwork, the more it is influenced by rewiring when creating the null networks. Fourth, wereproduced results in a second dataset of young adults (participants in HCP-D >18 yearsold).

The current study also has several limitations. First, edge-centric approaches square thenumber of elements compared with node-centric approaches. As such, E-NCT requires comparativelymore memory and computational resources than node-centric NCT. The AAL2 atlas was chosen overother parcellations due to the lower number of nodes and associated reduced computational time.Computational complexity for E-NCT grows at$O(n^{2})$. Currently, calculating eAC and eMC takes ~150 s, while calculating controlenergy takes ~1.2 hours for each subject. Similar results were observed when using the Dosenbach160 node atlas (Dosenbach et al., 2010), suggesting E-NCTis not atlas dependent. Second, it may be difficult to generalize E-NCT resting-state fMRI data.Functional connectivity includes positive and negative correlations between regions depending onpreprocessing choices (Murphy & Fox, 2017;Murphy et al., 2009). However, “negative” edgeweights are difficult to conceptualize in NCT. Future work may focus on silencing brain activityto activate a “negative” brain network (Tu et al.,2021), parallel to stimulating brain regions to activate a positive one. InvestigatingE-NCT with resting-state fMRI and associations with various processing choices (e.g., globalsignal regression) is future work. Third, to fully model the information flow within structuralnetworks, it is necessary to know the axonal directionality or theoretically define thedirection of edges in the network. Diffusion MRI used in most human studies can quantify thestrength of the structural connection but fail to provide information about the direction ofinformation flow. Future research should incorporate directional information into NCT. Fourth,we formulate E-NCT with different settings (discrete or continuous) based on the measures. Weuse the discrete setting for eAC and eMC and the continuous setting for control energy. Thisapproach was chosen to be consistent with the papers that introduced these measures inneuroimaging. Future work is needed to compare E-NCT under the two settings.

## 5 Conclusion

In conclusion, we proposed an E-NCT to explore the capability of each edge of the structuralconnectome in supporting brain state transitions. Our findings show that edge controllabilityprovides a complementary measure of NCT. By considering the interactions between edges, E-NCTnaturally distributes a node’s controllability among its edges, distinguishing betweenwithin- and between-network effects and giving a more comprehensive view of brain dynamics. Insum, E-NCT provides an edge-centric perspective on the brain’s network controlmechanism.

## Supplementary Material

Supplementary Material

## Data Availability

The Human Connectome Project (HCP) dataset is publicly available athttps://db.humanconnectome.org/with theacceptance of HCP Open Access Data Use Terms. The diffusion data were processed with DSI studiohttps://dsi-studio.labsolver.org/andfunctional data were processed withhttps://bioimagesuiteweb.github.io/. The original edge-centric network control theorycode is publicly available athttps://github.com/huiliii/edge_control.
