# Supplementary material for: Edge-centric network control on the human brain structural network
Source: Imaging Neurosci (Camb). 2024 Jun 10;2:imag-2-00191. doi: 10.1162/imag_a_00191 (PMC12272207; doi:10.1162/imag_a_00191)
Supplement: Supplementary Material [file imag_a_00191-supp.pdf]

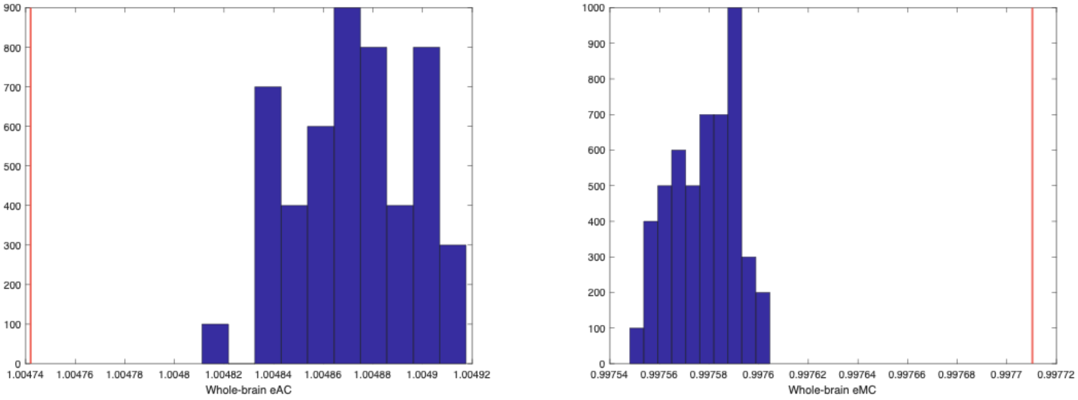

Fig. S1. Edge controllability of null models. Using nonparametric tests, the empirical whole-brain edge average controllability is lower than those based on null models ($p<0.001$); the empirical whole-brain edge modal controllability is higher than those based on null models ($p<0.001$).

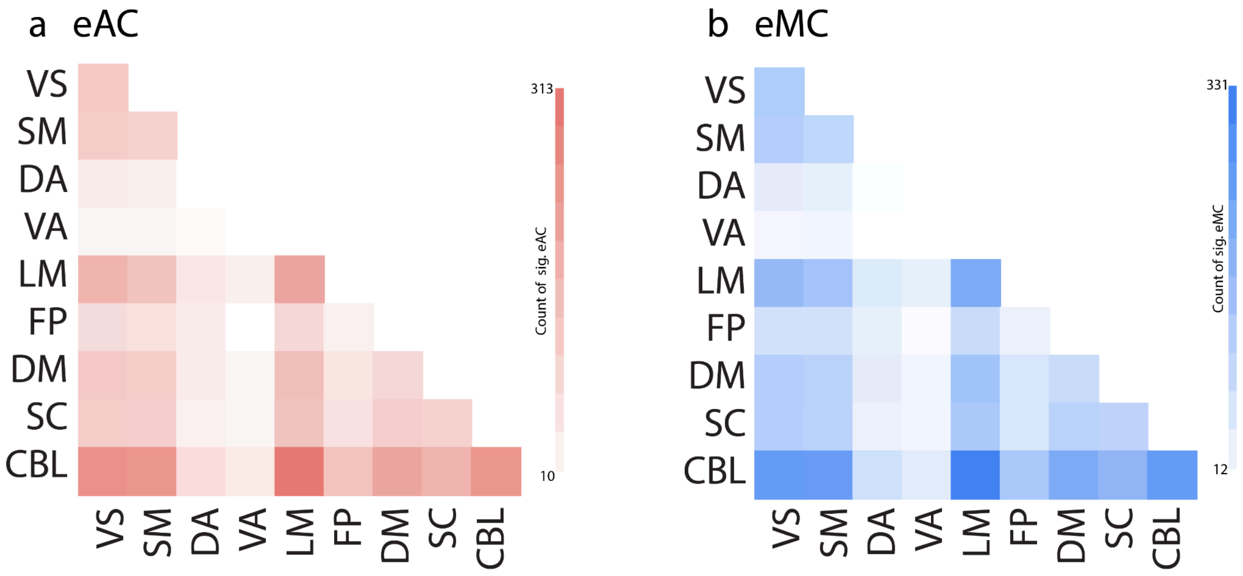

Fig. S2. Edge controllability of null models in canonical networks. Among 5000 null models, the heat maps show the number of edges with significant (a) lower average controllability and (b) higher modal controllability. VI=visual, SM=somatomotor, DA=dorsal attention, VA=ventral attention, LM=limbic, FP=frontoparietal, DM=default mode, SC=subcortical, and CBL=cerebellar networks

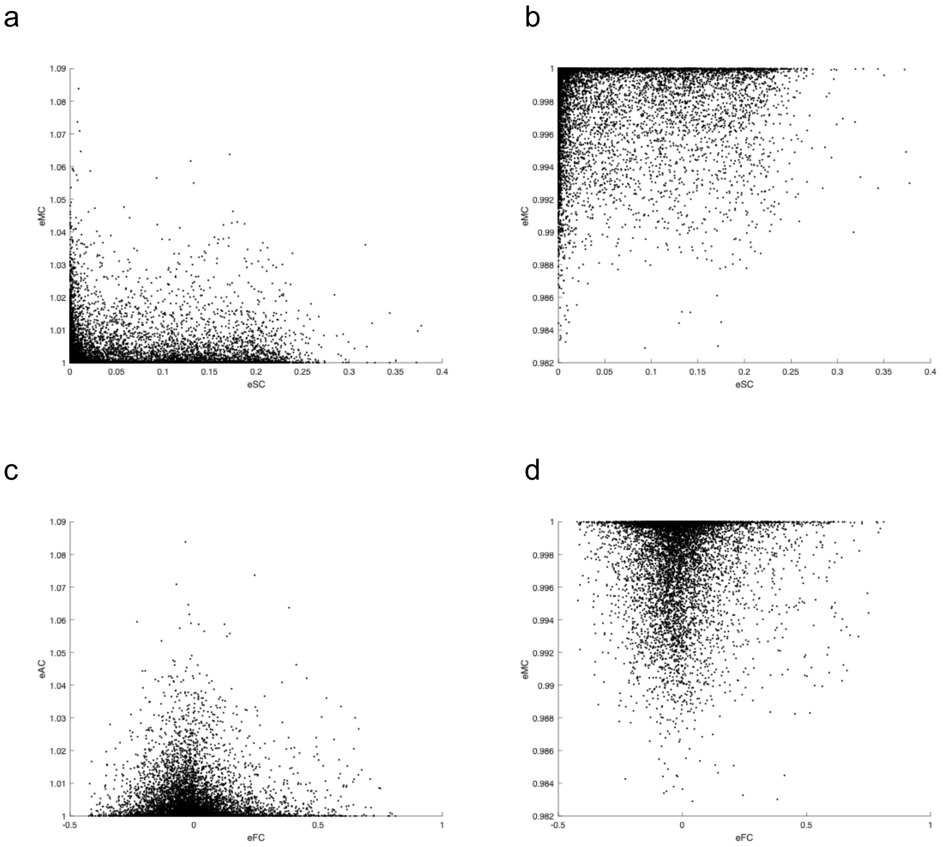

Fig. S3. Scatter plots of the association between edge-level average and modal controllability and SC/FC.

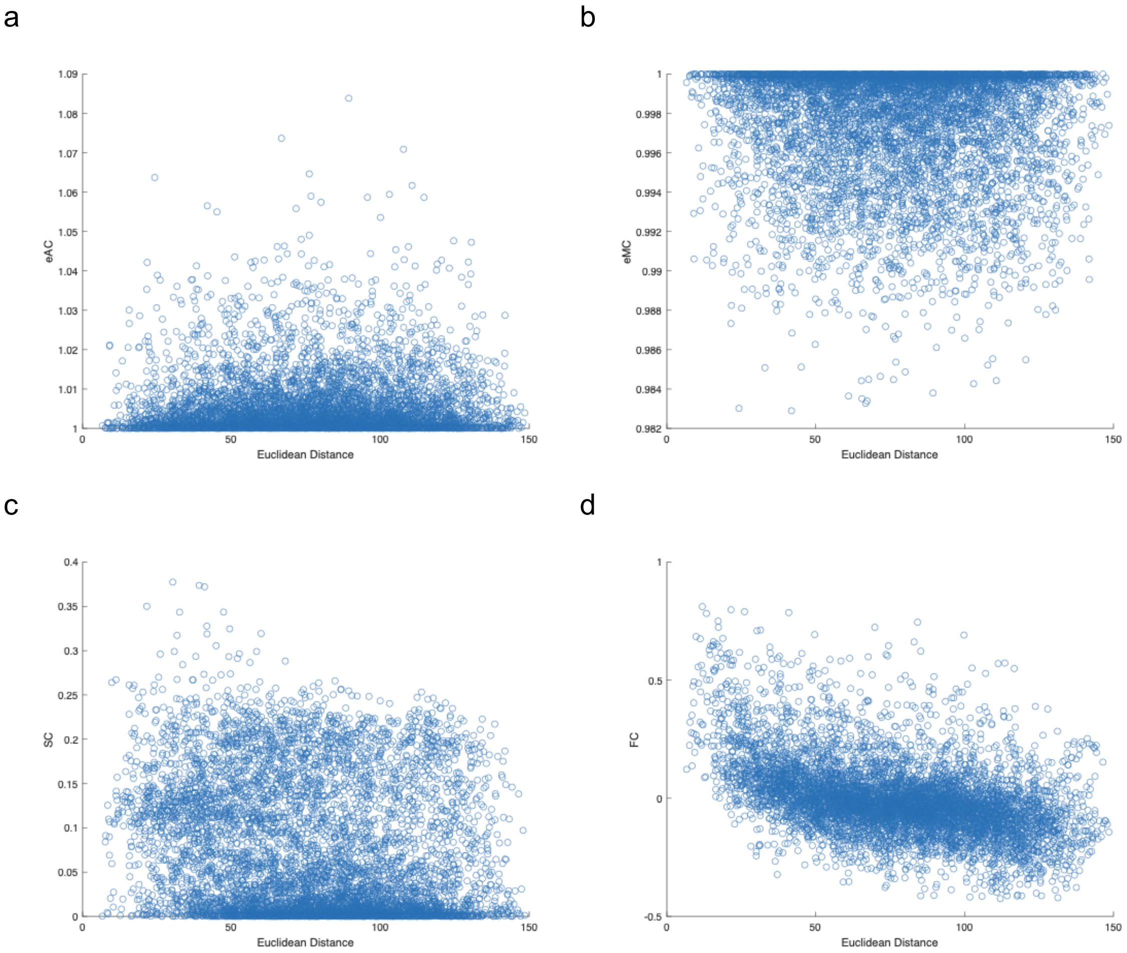

Fig. S4. Scatter plots of the associations between the Euclidean distance and eAC(a)/eMC(b)/SC(c)/FC(d).

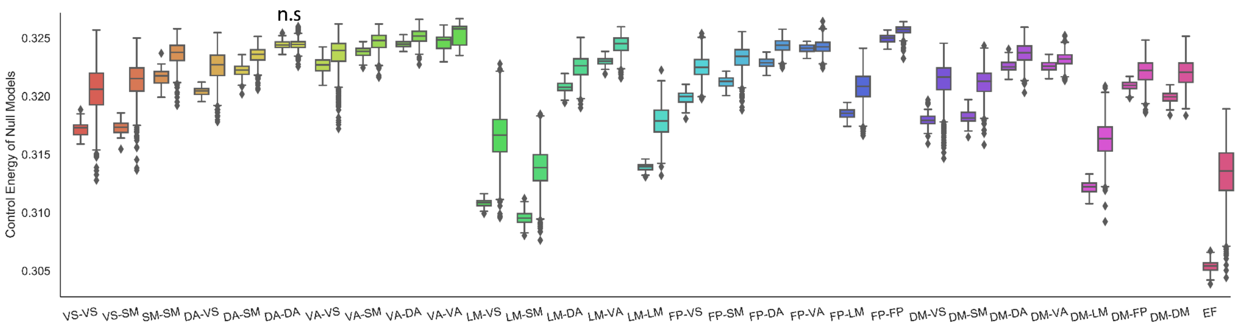

Fig. S5. Normalized control energy needed to change connectivity in 28 canonical network pairs (7 within- and 21 between-networks) from the empirical brain networks (boxes on the right) and those of null models (boxes on the left). For each type of activation, the left box shows the control energy of activating the null models created from the group-averaged brain structural connectome preserving weight, degree, and strength distribution, and the right box shows the control energy of the empirical structural network. The control energy of null models is significantly lower than that of real brain networks in all types of activation except for edges within the dorsal attention network.

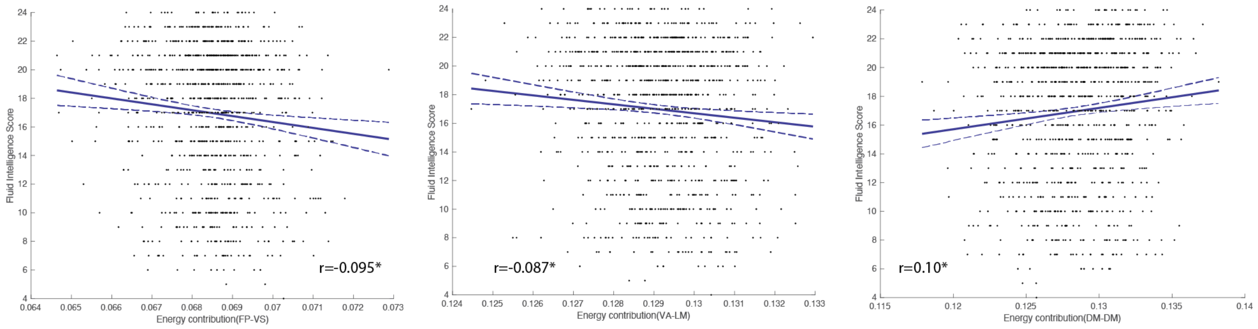

Fig. S6. Associations between individual differences in network-level energy contributions for the EF network and their fluid intelligence performance. The energy contributions of FP-VS (r=-0.095 , p=0.028, FDR-corrected) and VA-LM (r=-0.087, p=0.045, FDR-corrected) were negatively correlated with individual fluid intelligence scores, while those of DM-DM were positively correlated with intelligence (r=0.10, p=0.024, FDR-corrected).

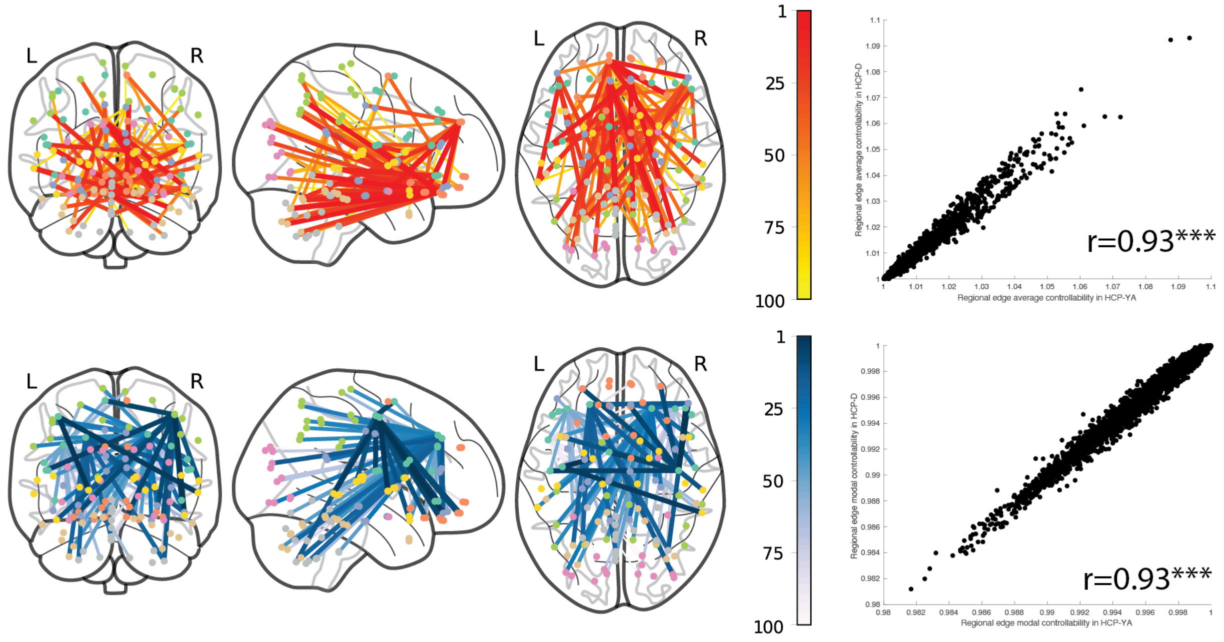

Fig. S7. Edge controllability distribution among the HCP-D adult group. (a) Edges with Top 100 mean edge average (red) and modal (blue) controllability for subjects with age >18 years in the HCP-D dataset. (b) Mean edge controllability in HCP-YA and that in HCP-D is highly aligned (Pearson's correlation: mean edge average controllability: r=0.9323, p=0; mean edge modal controllability ; r=0.9260, p=0).

**Table S1.** Demographic characteristics of subjects in HCP-D (n=149)	
Sex, num of females (%)	81(54%)
Age/weeks (mean(std))	241.11(13.94)

**Table S2.** Correlations between observed and predicted EF scores, IQ, and age. Reported values represent the median (r,p) value over 1000 iterations.						
	Executive Function				IQ	Age
	Card Sorting	Flanker	List Sorting	EF-PCA1		
Node AC+MC	0.0041 (0.50)	0.040 (0.19)	0.11 (<0.001)	0.021 (0.40)	0.11 (<0.001)	0.037 (0.23)
Edge AC	0.018 (0.47)	0.087 (0.0045)	0.13 (<0.001)	0.045 (0.14)	0.12 (<0.001)	0.12 (<0.001)
Edge MC	0.035 (0.34)	0.12 (<0.001)	0.16 (<0.001)	0.080 (0.0089)	0.16 (<0.001)	0.18 (<0.001)
SC	0.018 (0.47)	0.086 (0.0051)	0.088 (0.0042)	0.074 (0.016)	0.088 (0.0040)	0.18 (<0.001)
FC	0.078 (0.079)	0.02 (0.44)	0.00 (0.54)	0.11 (0.018)	0.10 (0.022)	0.15 (<0.001)
Limited to 120 features						
Edge AC	0.018 (0.43)	0.086 (0.0046)	0.13 (<0.001)	0.038 (0.21)	0.12 (<0.001)	0.12 (<0.001)
Edge MC	0.044 (0.15)	0.11 (<0.001)	0.15 (<0.001)	0.081 (0.0085)	0.16 (<0.001)	0.18 (<0.001)
SC	0.025 (0.37)	0.086 (0.0052)	0.083 (0.0065)	0.071 (0.020)	0.079 (0.011)	0.17 (<0.001)
FC	0.081 (0.067)	0.030 (0.45)	0.0081 (0.45)	0.0040 (0.60)	0.027 (0.50)	0.15 (<0.001)

**Table S3**. Correlations between observed and predicted EF scores, IQ, and age. Reported values represent the median r-value over 100 iterations using kernel ridge regression						
	Executive Function				IQ	Age
	Card Sorting	Flanker	List Sorting	EF-PCA1		
Node AC+MC	0.047	0.00066	0.12	0.029	0.13	0.19

Edge AC	0.010	0.039	0.17	0.039	0.16	0.20
Edge MC	0.070	0.065	0.15	0.032	0.15	0.25
SC	0.090	0.064	0.15	0.041	0.19	0.29

Table S4. Associations between edge controllability and structure/functional connectomes in HCP-D

	eAC	eMC
nAC	r=0.93<br>p=2.53e-53	/
nMC	/	r=0.98<br>p=1.04e-85
SC-node	r=0.89<br>p=3.75e-42	r=-0.99<br>p=1.01e-99
SC-edge	r=-0.05<br>p=1.20e-5	r=0.05<br>p=9.87e-6
FC-node	r=-0.41<br>p=2.95e-6	r=0.44<br>p=4.61e-7
FC-edge	r=-0.0011<br>p=0.93	r=0.011<br>p=0.34
